# A Recurrent Cheek Mass

**DOI:** 10.7759/cureus.31300

**Published:** 2022-11-09

**Authors:** Rita Makhoul, Sammy Tawk, Fouad El Sayed

**Affiliations:** 1 Dermatology, Universite Catholique de Louvain, Namur, BEL; 2 Radiology, Universite Catholique de Louvain, Namur, BEL; 3 Dermatology, Lebanese University - Faculty of Medicine, Beirut, LBN

**Keywords:** targeted therapy, surgery, recurrence, myofibromatosis, solitary infantile myofibroma

## Abstract

Solitary infantile myofibroma is a benign fibrous tumor occurring in early childhood. Although rare, it is the most common benign fibrous tumor of infancy. The clinical course of the disease is almost uniformly good since most tumors regress spontaneously. When indicated, conservative surgical excision is the treatment of choice, with a low recurrence rate. We present a case of solitary infantile myofibroma that recurred after three attempts of surgical excision, questioning the reported recurrence rate and the standard of care in recurrent solitary infantile myofibroma.

## Introduction

Infantile myofibromatosis (IM) is a mesenchymal disorder characterized by myofibroblastic proliferation that results in solitary, multicentric, or generalized neoplasms affecting the skin, bone, and viscera [1]. It was first described by Stout in 1954 in its congenital generalized form. For a long time, fibromatoses were known as locally invasive, aggressive, and multicentric diseases [2]. Later on, solitary forms were described and the term “infantile myofibromatosis” was adopted by Chung et al. given the occurrence of these lesions in both newborns and infants [2].

Although rare, IM is considered the most common benign fibrous tumor in infancy [3]. It is typically diagnosed in young children, usually before the age of two, with a wide range of clinical spectrums, ranging from a solitary asymptomatic soft tissue nodule to disseminated tumors with life-threatening complications, especially when associated with visceral involvement [3].

Gain-of-function mutations of platelet-derived growth factor receptor-beta (PDGFRB) were found in affected children, especially those affected by the multicentric form of IM, albeit present in solitary IM [3]. In addition, a novel COL4A1-VEGFD fusion transcript was identified as a recurrent genetic event. These mutations shed light on a pathogenic mechanism that can be aimed at by targeted therapies and could replace surgeries and chemotherapy-based treatment [3].

## Case presentation

**Figure 1:**
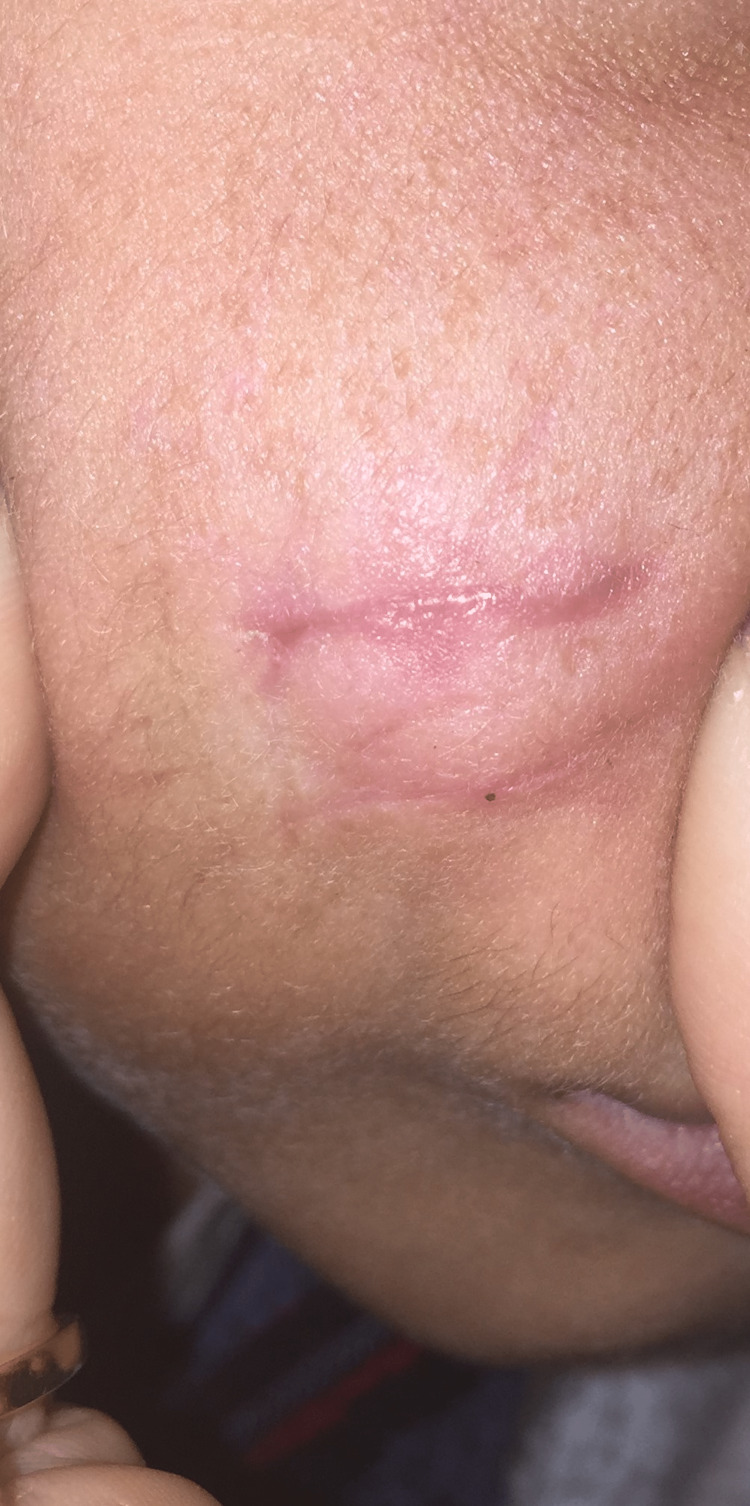
Clinical image showing the scars of previous surgeries and the firm, round, subcutaneous tumor

**Figure 2:**
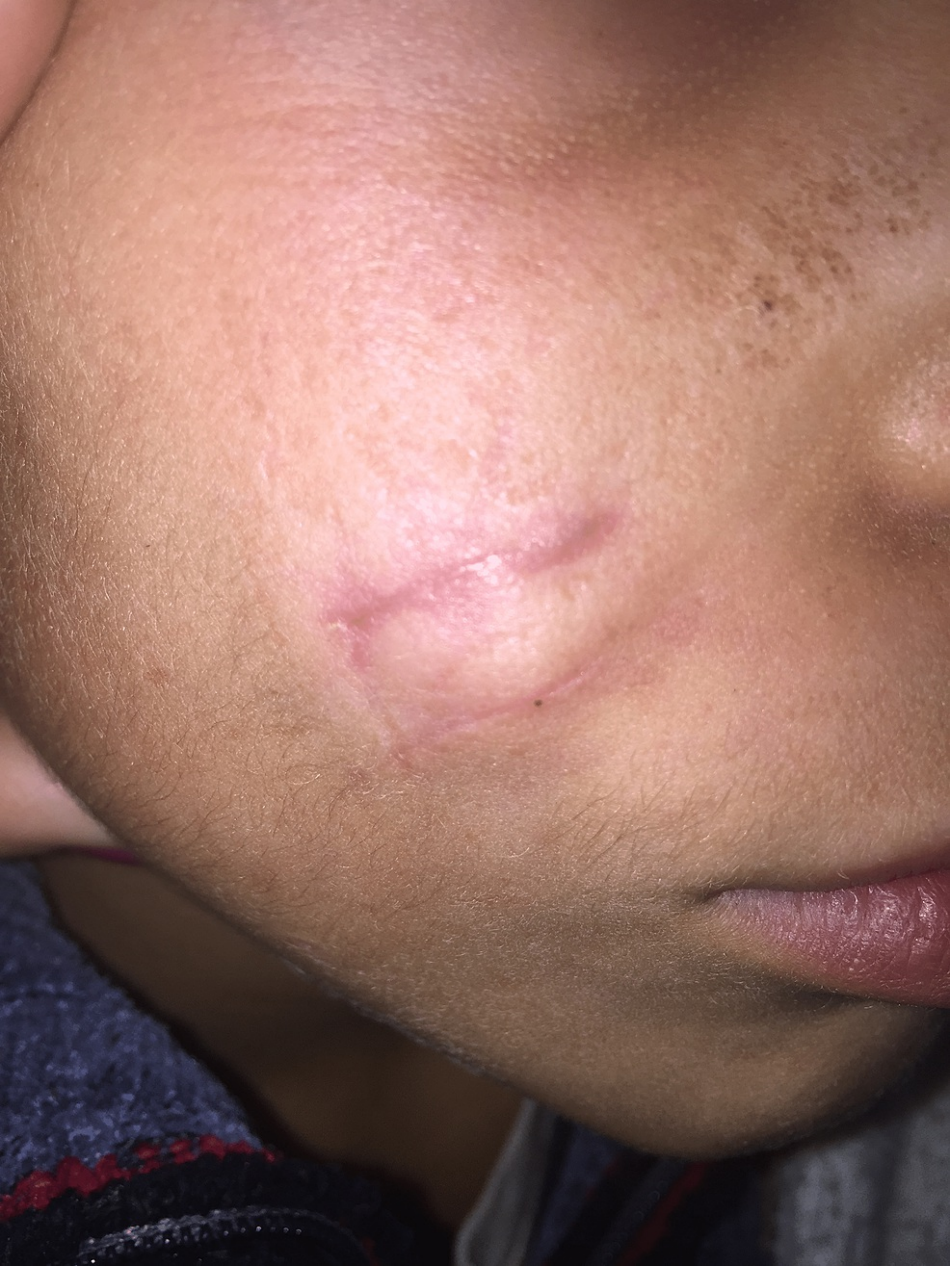
Clinical image showing the scars of previous surgeries and the firm, round, subcutaneous tumor

A 10-year-old healthy boy presented to our dermatology department for a right cheek nodule. It appeared for the first time during early infancy. It was excised twice and recurred each time two to three months after its excision. The lesion was asymptomatic and didn’t generate any mass effect or physical limitation. The family history was unremarkable. On physical exam, two scars were identified on the right cheek and a firm, round, partially mobile, 3 cm subcutaneous nodule was palpated (Figures 1, 2).

Based on history, clinical findings, and complete physical exam, the differential diagnosis included infantile fibrosarcoma, neurofibroma, dermoid, and desmoid tumors.

**Figure 3:**
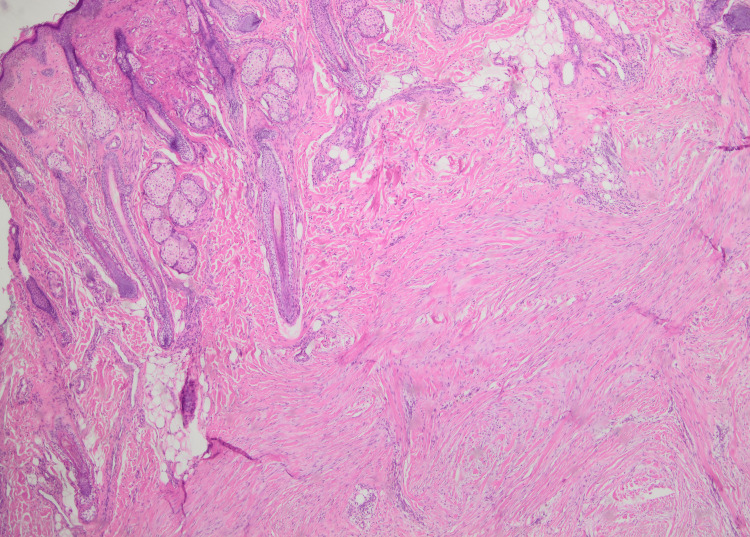
Histological image showing a nodular tumoral proliferation in the reticular dermis and fusiform biphasic cells arranged in fascicles

**Figure 4:**
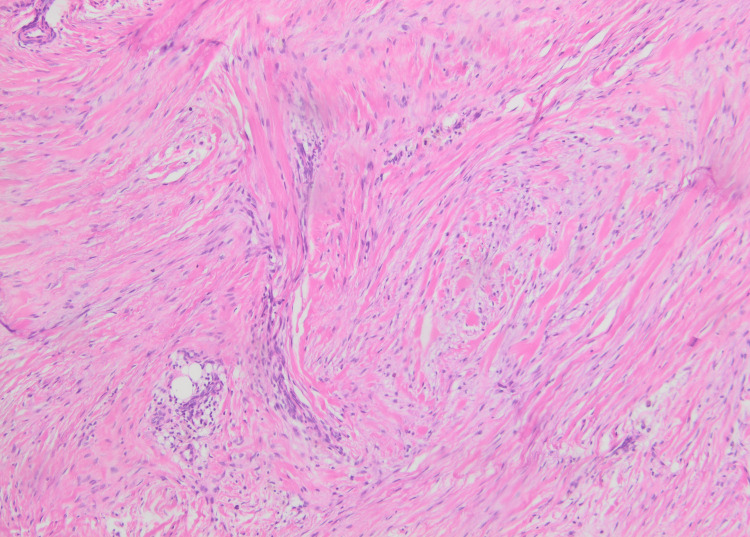
Histological image showing a nodular tumoral proliferation in the reticular dermis and fusiform biphasic cells arranged in fascicles

Biopsy was taken and histopathology showed nodular proliferation in the reticular dermis composed of fusiform biphasic cells arranged in fascicles in a mixed (pale myxoid/hyalinized) stroma without pleomorphism or atypia. These findings were consistent with a myofibroma (Figures 3, 4).

**Figure 5:**
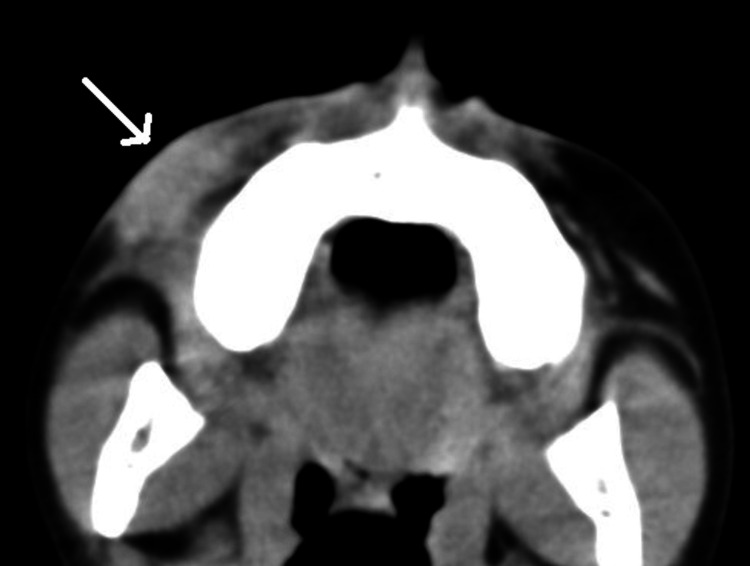
Enhanced CT scan of the face showing an enhancing subcutaneous soft tissue lesion of the right cheek (arrow)

**Figure 6:**
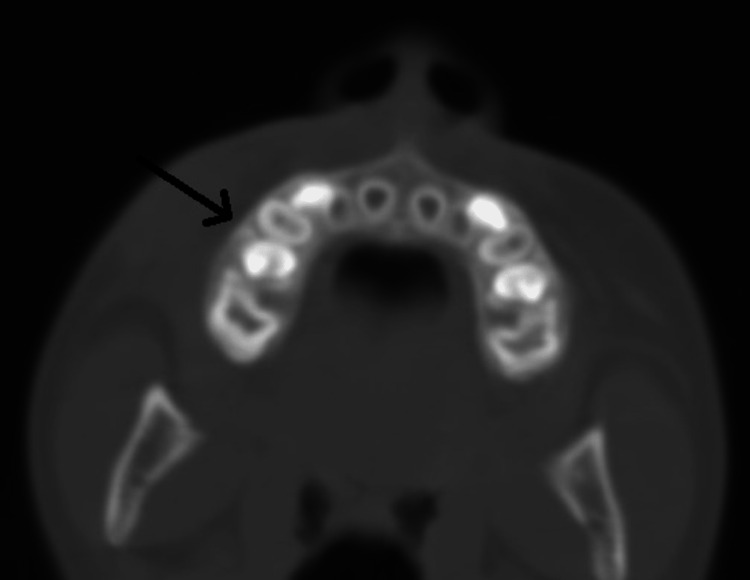
Enhanced CT scan of the face showing an intact underlying cortical bone (arrow)

Computed tomography (CT) of the face showed that the myofibroma affected the skin and subcutaneous tissue without bony involvement (Figures 5, 6).

To rule out further tissue involvement, a CT scan of the chest, ultrasound of the abdomen, and X-ray of long bones were performed and came back negative.

The tumor was excised based on the parents’ decision despite our advice for conservative management. Excision was complete with tumor-free margins on histopathology and the diagnosis of IM was re-confirmed based on the presence of fusiform biphasic cells arranged in fascicles in a mixed stroma. Unfortunately, the myofibroma recurred, once again, only a few months after its surgical removal.

## Discussion

Infantile myofibromatosis/myofibroma is a rare fibrous disorder of childhood and infancy, its incidence ranges between 1 in 150,000-400,000 [4]. Most cases are diagnosed before the age of two, with 60% of them present at or soon after birth [5], and a slight male predominance [6]. IM is char­acterized by the development of tumors involving the skin, subcutaneous tissue, bones, or viscera [1]. Different forms of IM were described: the solitary infantile myofibroma, the multicentric form, and the generalized form with visceral involvement. All subtypes share similar histopathological findings but have different clinical features and prognoses [6].

Clinically, IM presents as one or more rubbery to firm nodules. Lesions are usually asymptomatic, skin-colored to purple, and located in the dermis or subcutaneous tissue with the possibility of muscular, skeletal, and visceral involvement [2,7]. The head and neck are the most commonly affected regions, followed by the trunk [8]. Our patient had a typical presentation with a solitary infantile myofibroma that first appeared during early infancy and was limited to the subcutaneous tissue of the cheek, sparing mandibular bones.

Histopathologically, IM is described as a well-circumscribed, non-capsulated mass characterized by a distinctive biphasic growth pattern. The center of the lesion is composed of immature, plump, myofibroblastic, spindled tumor cells with hemangiopericytoma-like branching blood vessels while the periphery consists of nodules and fascicles of hyalinized, myoid-appearing cells. Despite the impression of circumscription, IM sometimes demonstrates infiltrative growth. Note that myofibroma and myopericytoma share some features, suggesting that these entities could be related [9]. Immunohistochemically, IM usually stains positive for smooth muscle actin and vimentin while it stains negative for desmin [6,8].

In their review of the literature, Braun et al. found that 35% of published cases had solitary cutaneous or subcutaneous IM [10]. Of those with a single lesion, 9% were later found to have bone, muscle, and visceral involvement, and up to 5% experienced morbidity or mortality (1%) related to extracutaneous involvement before undergoing full body screening [10]. Although there are no international published guidelines for tumor staging, various authors recommend screening patients for visceral involvement by chest X-ray, echocardiogram, and whole-body magnetic resonance imaging [5,6,10]. Serial screening for solitary cases remains controversial [10]. In our case, due to limited resources, we opted for a CT scan of the chest, an ultrasound of the abdomen, and an X-ray of long bones that ruled out skeletal and visceral involvement with their potential morbidity.

Familial cases have been reported without a clear inheritance pattern but our patient had no family history of IM [8]. Gain-of-function mutations of platelet-derived growth factor receptor-beta (PDGFRB) were found in affected children, especially those with multicentric IM, albeit present in solitary IM [3]. In addition, a novel COL4A1-VEGFD fusion transcript was identified as a recurrent genetic event in patients with IM, shedding light on a pathogenic mechanism that might be an aim for targeted therapies [3].

Patients may experience morbidity related to growth retardation, vocal cord paralysis, pathologic fractures, and physical limitations due to the mass effect [10]. In vertebral IM, pathologic fractures might affect the spinal cord [10]. On the other hand, visceral involvement in the generalized form is associated with a poorer outcome and a mortality rate reaching 76% [6,11,12]. The total mortality rate for all patients with IM is around 4.5%, with cardio-respiratory failure, sepsis, pulmonary hemorrhage, gastrointestinal obstruction, and cerebral hemorrhage being the main reported causes of death [10].

An initial increase in the number and size of the lesions was frequently reported, but the majority of IM cases (in the solitary as well as multicentric forms) follow the typical benign course with spontaneous regression in the first few years following the diagnosis. Different theories were postulated as explanations of this process but were never confirmed, in particular, the apoptotic mechanism and angiogenesis modulating factors [1,5,6,10].

Considering the frequent spontaneous regression, close observation remains the most appropriate approach in the majority of cases. When tumors affect vital functions or cause growth anomalies or cosmetic deformities, conservative surgical excision is the standard treatment [5,13,14]. In the case described, re-excision was performed despite advice to follow a more conservative approach, especially in the context of previous recurrences and absent invasive disease.

There are no definitive guidelines for generalized IM treatment. Low-intensity chemotherapy with vinblastine-methotrexate, vincristine- actinomycin-D, or interferon-alfa or antiangiogenic therapy (ie, tamoxifen) might be considered in this form despite their side effects and unconfirmed efficacy [5,6,15,16]. Previously, chemotherapy with or without radiotherapy has been used in patients with recurrent lesions [17]. Following the discovery of a gain of function mutation in PDGFRB, targeted therapy by imatinib and other tyrosine kinase inhibitors was supported by recent literature [18,19].

The reported recurrence rate of IM after excision is 7-10% [2,20]. While re-excision of recurrent lesions is usually curative, our patient was among those few cases where the tumor exceptionally recurred three times despite adequate surgical excision.

## Conclusions

Infantile myofibromatosis is a rare disease of infancy. When indicated, conservative surgery is the treatment of choice in solitary forms. Although IM seldom recurs, it recurred exceptionally after three attempts at surgical excision in our case, questioning the reported recurrence rate. Recurrent lesions could also be treated by chemotherapy with or without radiotherapy. Since recent literature supports targeted therapy by tyrosine kinase inhibitors, it could replace the standard aggressive treatments in primary IM as well as their recurrences. Further research is still required to assess their safety and efficacy.

## References

[1] Beck JC, Devaney KO, Weatherly RA, Koopmann CF Jr, Lesperance MM (1999). Pediatric myofibromatosis of the head and neck. Arch Otolaryngol Head Neck Surg.

[2] Chung EB, Enzinger FM (1981). Infantile myofibromatosis. Cancer.

[3] Dachy G, Fraitag S, Boulouadnine B, Cordi S, Demoulin JB (2021). Novel COL4A1-VEGFD gene fusion in myofibroma. J Cell Mol Med.

[4] Street CM, Hill SJ (2021). Solitary lung myofibroma in an infant. J Pediatr Surg Case Rep.

[5] Mahajan P, Venkatramani R (2020). Comment on: Solitary myofibroma preceding the development of multicentric myofibromatosis: a report of two cases with surveillance recommendations. Pediatr Blood Cancer.

[6] Mashiah J, Hadj-Rabia S, Dompmartin A (2014). Infantile myofibromatosis: a series of 28 cases. J Am Acad Dermatol.

[7] Stanford D, Rogers M (2000). Dermatological presentations of infantile myofibromatosis: a review of 27 cases. Australas J Dermatol.

[8] Bolognia JL, Jorizzo JL, Schaffer JV (2012). Dermatology, 3rd Edition. https://www.scholars.northwestern.edu/en/publications/dermatology-3rd-edition-2.

[9] Foss RD, Ellis GL (2000). Myofibromas and myofibromatosis of the oral region: a clinicopathologic analysis of 79 cases. Oral Surg Oral Med Oral Pathol Oral Radiol Endod.

[10] Braun M, Pascual M, Mully T, Phelps A, Prok L, Shah SD, Kohn LL (2022). Solitary cutaneous infantile myofibroma as a hallmark of myofibromatosis: two cases and review of the literature. Pediatr Dermatol.

[11] Jones VS, Philip C, Harilal KR (2007). Infantile visceral myofibromatosis--a rare cause of neonatal intestinal obstruction. J Pediatr Surg.

[12] Römer T, Wagner N, Braunschweig T, Meyer R, Elbracht M, Kontny U, Moser O (2021). Aggressive infantile myofibromatosis with intestinal involvement. Mol Cell Pediatr.

[13] Taguchi T, Matsuura T, Kinoshita Y (2018). Tumors of the Head and Neck. Rintala RJ, Hutson JM.

[14] Zhao G, Zhu M, Qin C, Liu X, Zhao X (2020). Infantile myofibromatosis: 32 patients and review of literature. J Pediatr Hematol Oncol.

[15] Gandhi MM, Nathan PC, Weitzman S, Levitt GA (2003). Successful treatment of life-threatening generalized infantile myofibromatosis using low-dose chemotherapy. J Pediatr Hematol Oncol.

[16] Auriti C, Kieran MW, Deb G, Devito R, Pasquini L, Danhaive O (2008). Remission of infantile generalized myofibromatosis after interferon alpha therapy. J Pediatr Hematol Oncol.

[17] Pattisapu P, Wenger TL, Dahl JP (2022). Avoidance of surgery for head and neck infantile myofibromatosis using imatinib monotherapy. Clin Case Rep.

[18] Bidadi B, Watson A, Weigel B, Oliveira A, Kirkham J, Arndt C (2020). Treatment of generalized infantile myofibromatosis with sorafenib and imatinib: a case report. Pediatr Blood Cancer.

[19] Mocellin S (2020). Myofibroma. Soft Tissue Tumors.

[20] Parker RK, Mallory SB, Baker GF (1991). Infantile myofibromatosis. Pediatr Dermatol.

